# An automated auroral detection system using deep learning: real-time operation in Tromsø, Norway

**DOI:** 10.1038/s41598-022-11686-8

**Published:** 2022-05-31

**Authors:** Sota Nanjo, Satonori Nozawa, Masaki Yamamoto, Tetsuya Kawabata, Magnar G. Johnsen, Takuo T. Tsuda, Keisuke Hosokawa

**Affiliations:** 1 Graduate School of Informatics and Engineering, University of Electro-Communications, Chofu, 182-8585 Japan; 2 Institute for Space-Earth Environmental Research, Nagoya University, Nagoya, 464-8601 Japan; 3 Canon Inc., Information and Communication Systems Headquarters, Ota, 146-8501 Japan; 4 UiT The Arctic University of Norway, Tromsø Geophysical Observatory, 9037 Tromsø, Norway

**Keywords:** Aurora, Software

## Abstract

The activity of citizen scientists who capture images of aurora borealis using digital cameras has recently been contributing to research regarding space physics by professional scientists. Auroral images captured using digital cameras not only fascinate us, but may also provide information about the energy of precipitating auroral electrons from space; this ability makes the use of digital cameras more meaningful. To support the application of digital cameras, we have developed artificial intelligence that monitors the auroral appearance in Tromsø, Norway, instead of relying on the human eye, and implemented a web application, “Tromsø AI”, which notifies the scientists of the appearance of auroras in real-time. This “AI” has a double meaning: artificial intelligence and eyes (instead of human eyes). Utilizing the Tromsø AI, we also classified large-scale optical data to derive annual, monthly, and UT variations of the auroral occurrence rate for the first time. The derived occurrence characteristics are fairly consistent with the results obtained using the naked eye, and the evaluation using the validation data also showed a high F1 score of over 93%, indicating that the classifier has a performance comparable to that of the human eye classifying observed images.

## Introduction

The contribution of citizen scientists has recently been highlighted in the field of the space science. Strong Thermal Emission Velocity Enhancement (STEVE) is an optical emission in the upper atmosphere of the polar region, which was newly discovered by citizen scientists using digital cameras^1,2^. The apparent shape of STEVE looks like aurorae, more often appearing in the same altitudes due to the precipitations of electrons from the near-Earth space. However, the observations made using digital cameras by citizen scientists and other related professional observations demonstrated that the spectral characteristics of STEVE are completely different from those of typical greenish aurorae and that it should thus, be recognized as a new atmospheric phenomenon produced via the heating of the atmosphere by a fast plasma flow (i.e., not caused by the precipitation of auroral electrons). In another example, citizen scientists have observed unusual wavy aurorae called “dunes” from several locations in the polar region simultaneously. Such multi-point observations have enabled the accurate estimation of the emission altitude of $\sim$100 km^3^. This suggests that dunes are mainly modulated by atmospheric gravity waves. Digital cameras not only help ascertain the shape and geographic location of atmospheric optical phenomena but also their color. Although the color is not a physical quantity, it was recently indicated that the colors of pulsating auroras, which is one of the major categories of the diffuse-type auroras^4^ could be qualitatively related to the energy of electrons^5^. Thus, observations made using digital cameras will play a meaningful role in gaining a more detailed understanding of the cause and nature of aurorae and related atmospheric phenomena. However, since the auroral hunters have to wait for the appearance of auroras in the field outside under the cold weather, it is still not easy for them to participate in research space science by taking photographs. In order to reduce such a heavy workload, professional researchers are required to provide information on how the occurrence of auroras depends on the background conditions (e.g., year, month, and local time) and further launch a notification service of the auroral activity in real-time.

The occurrence of auroras has been studied with the naked eye since the 18th century. The annual and seasonal variations in the occurrence of auroral displays were investigated using 1000-time naked-eye observations at two sites in southern Finland over a 100-year period from 1748^6^. According to this study, the auroral occurrence has an 11-year cycle, similar to the solar activity, and a few auroras were observed during the Dalton Minimum, which is one of the periods of the lowest solar activity^7^. They also suggested that similar to the geomagnetic storms, the occurrence rate of auroras is higher in autumn/spring, and lower in summer/winter. However, since they observed the aurora without any optical instruments, the statistical significance of their results is relatively low. Another study has visually classified images observed by all-sky cameras (ASCs) to derive the occurrence rate of auroras^8^. They captured images for 100,000 h using ASCs in Finland and Svalbard over a 24-year period from 1973 and derived the local time dependence of the auroral occurrence rate. Similar studies using ASCs in other high-latitude regions were conducted later, and a common tendency that was observed was that the auroral occurrence peaked just before/after the magnetic midnight^9–11^. However, classifying data with the naked eye is physically tough and time-consuming, and it is difficult to apply such an approach to analyze recent data, which have a much higher temporal resolution.

To classify the current large-volume optical data, it is more efficient to use an automated classification performed via artificial intelligence (AI), rather than using the human eye. Automated classification techniques for auroral images using hidden Marcov models^12^, k-nearest neighbor (kNN)^13,14^ and support vector machines (SVMs)^15^ had been developed in the last decade. To further improve an accuracy, classification methods combining multiple algorithms were proposed and applied to the dayside auroras^16–18^. However, these algorithms have been difficult to be used casually because their accuracy depends on manually selected feature values. This drawback was overcome by the advent of the deep-learning technique, which automatically selects such values. A method for classifying daytime auroras using the deep neural network (DNN) was then proposed^19^, but this method was not used for conducting a statistical analysis of the auroral occurrence because it cannot classify non-auroral images. Later, a study that classified gray-scale all-sky images including non-auroral images using DNN^20^ performed the classification with 82% accuracy; the images were classified into six classes: *Arc*, *Discrete*, *Diffuse*, *Cloudy*, *Moon*, and *Clear*. The authors of this study also suggested using other classes such as *auroral activity with moon contamination*, but whether it could show a high accuracy and yield classes that were more complex than the six example classes was not tested. Real-world optical data often include noisy auroral images, such as those with clouds, moonlight, and sunlight contamination. Any ambiguity in the definition of the auroral image hampers the automatic detection and the statistical analysis of the auroral occurrence. Therefore, it is important to distinguish the quality of the optical data, regardless of whether it is just an auroral image. A similar study using color all-sky images demonstrated that a DNN model called ResNet-50^21^ can classify the images into seven auroral classes with an average precision and F1 score of 92% and 90%, respectively^22^. Here, precision and recall are the ratios of true positive (TP) to TP + false positive (FP) and that of TP to TP + false negative (FN), respectively. The F1 score is a harmonic mean of precision and recall. However, because the authors of this previous study did not train non-auroral images, such as those captured during cloudy conditions, it is still difficult to classify regularly captured color all-sky images and then perform a statistical analysis of the auroral occurrence rate. Thus, it is highly demanded to evaluate if machine-learning-based automated classification is sufficiently accurate for investigating the statistical characteristics of aurora such as its seasonal and local time distribution. It is also needed to test the feasibility of operating a notification system for the auroral appearance in a real-time manner.

**Figure 1:**
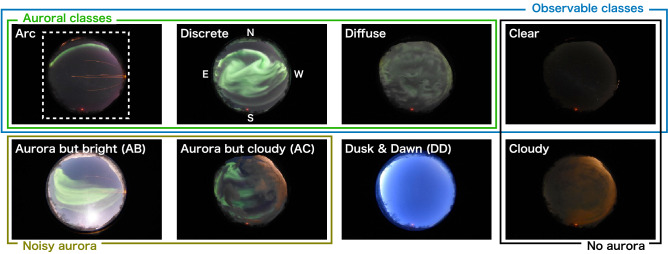
Sample images of the eight classes for the differentiation of all-sky images. The white dashed square in the image of the *Arc* class indicates the region used for training. For statistical analysis, we defined the auroral occurrence rate as the ratio of the *Auroral* classes (*Arc, Discrete, and Diffuse*) to the *Observable* classes (*Auroral* + *Clear*). *Noisy aurora* and *No aurora* classes are used in the bottom panel of Fig. 3.

In the current study, we automatically derived the yearly, monthly, and universal time (UT) variations of the auroral occurrence rate for the first time by classifying images captured over a period of 10 years using digital cameras in Tromsø, Norway ($69.6^{\circ }\,\hbox {N}$, $19.2^{\circ }\,\hbox {E}$). Refer to the Methods for details regarding the observation. We manually classified some of the observed images into eight classes, as shown in Fig. 1, and then, the ResNet-50 model trained them. As described in a previous study^20^, we defined *Arc*, *Discrete*, and *Diffuse* as the classes of auroras that are not contaminated by moonlight or clouds and have a high quality. The *Arc* is a single or double stripe extending in the east-west direction, often observed before midnight as a precursor to auroral breakup^23^. *Discrete* auroras are bright and curly auroras that can appear white or pink at the lower limit when they are particularly intense. The *Diffuse* aurora glows faintly and is mainly observed after midnight. The majority *Diffuse* is a pulsating aurora (PsA) that shows quasi-periodic variations in its brightness, i.e., variations ranging from a few to several tens of seconds^4,24^. We also defined *Clear* as the time when no aurora was detected, even though any type of auroras were observable without the influence of moonlight or clouds. In the statistical analysis, these four classes were collectively referred to as the *Observable* classes, and only these classes were used to calculate the auroral occurrence rate. The definition of the auroral occurrence rate is the number of *Auroral* classes divided by the number of images of the *Observable* classes. To distinguish between the quality of the optical data, we defined *Noisy aurora* as the auroral images contaminated by moonlight or sunlight (*Aurora but bright*) and obscured by clouds (*Aurora but cloudy*). We have also added classes for the cloudy and dawn/dusk images. Although we can identify the *Dusk & Dawn* images by calculating the solar elevation angle, we prepared a specified class for them to simplify the overall workflow. A more detailed description of each class is given in the “Methods” section. In addition to the statistical analysis, we also introduce a real-time notification system for the auroral appearance using the classifier. If citizen scientists can use this system to quickly catch the appearance of aurora, they may be able to take more photographs with less effort. Therefore, this system will allow more citizen scientists to participate in research regarding space science more efficiently.

## Results

### Training of ResNet-50

**Figure 2:**
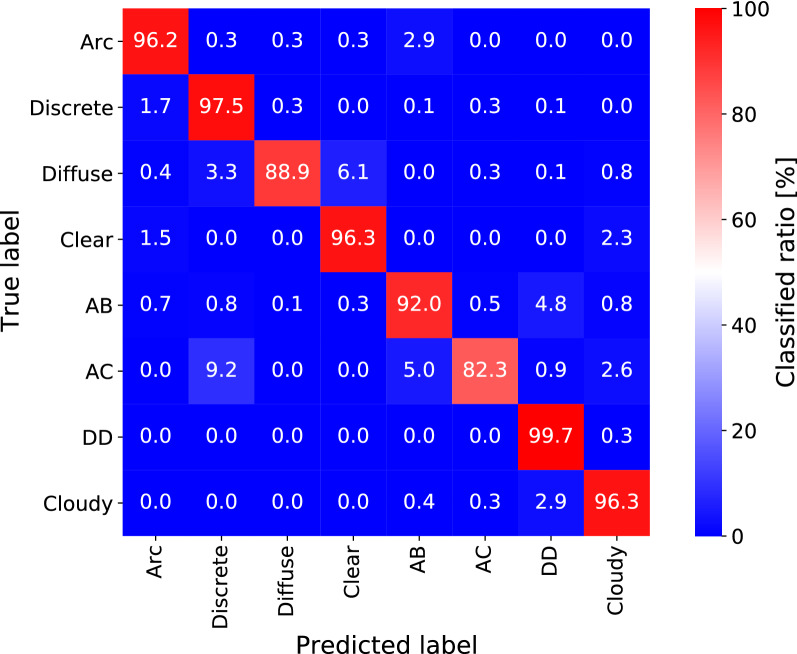
The classification result of the validation dataset by the trained ResNet-50 model. The top row shows that 96.2% of the 315 manually classified *Arc* images were correctly classified as *Arc*, while 2.9% were incorrectly classified as *Aurora but bright*. All the panels with matching true and predicted labels have high values of classified ratio, indicating high classifier accuracy.

A total of 5530796 images were captured in Tromsø from 2011 to 2021 season, of which 87275 (1.6% of the data) were manually labeled and then used for the training and evaluation of the ResNet-50 model. The images were also manually divided into two categories, i.e., training and validation, for each class, as shown in Table 1. Because the inclusion of similar images in both the categories leads to an unreasonably high accuracy, we ensured that no images observed on the same day were included in both the categories. Figure 2 shows the confusion matrix that exhibits the classification results of the trained ResNet-50 model against the validation data. All the validation images were allocated to a class having the largest probability. The values in each panel show the classification rate for each class. For example, the top-left cell shows that out of 315 *Arc* images in the validation data, 96.2% were correctly classified as *Arc* by the trained classifier. The panels where the true label and predicted label coincide, which are correctly classified cases, are all reddish, indicating that the classification is highly accurate. The average precision and F1 score were 93.1% and 93.4%, respectively. Since one of the purposes of this study is to detect auroras automatically, it is particularly important to ascertain whether an image belongs to an *Auroral* class. For distinguishing whether an image belongs to an *Auroral* class, the precision and F1 score should be even higher, at 97.1% and 95.9%, respectively.

**Table 1:** Number of images per class used for training and validation of the model.

Label	Arc	Discrete	Diffuse	Clear	AB	AC	DD	Cloudy
Training	2352	3516	7114	7764	4309	2880	23,599	20,680
Validation	315	766	2133	1553	862	576	4720	4136

Training and validation do not include images obtained on the same day.

**Figure 3:**
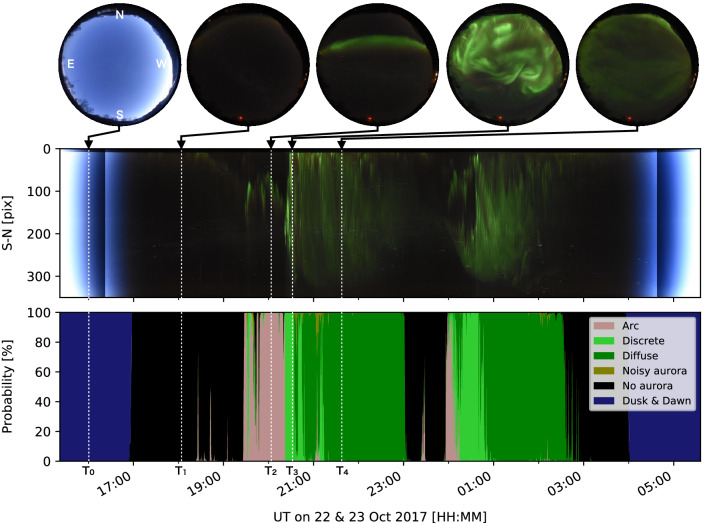
Observation and classification results from the night of 22–23 October 2017. The middle panel shows a keogram, which is a time series of the north-south cross-section of the all-sky image, and the bottom panel is the classification result for the same period. All-sky images at certain specific times ($\hbox {T}_0$ to $\hbox {T}_4$) are displayed on the top. $\hbox {T}_0$ to $\hbox {T}_4$ are 16:00:01, 18:00:05, 20:04:39, 20:30:40, and 21:30:11 UT, respectively. From 19:30 to 23:00 UT, the typical substorm, characterized by a sequential appearance of *Arc*, *Discrete*, and *Diffuse*, was almost perfectly classified. A similar substorm occurred again from midnight and was also classified correctly.

Figure 3 shows the observation and classification results for images from a night that was not used for either training or validation. On this night, a typical substorm, which comprised the *Arc*, *Discrete*, and *Diffuse* auroras, was observed twice. The format of Fig. 3 follows that of a previous study about the automatic detection of auroras^20^. The top row shows the all-sky images at several representative times ($T_0$ to $T_4$), the second row is the time-series of the north-south cross-section of the all-sky image (so-called keogram), and the third row shows the classification probability of each class. Since $T_0$ is shortly after sunset, the all-sky image and keogram are bluish. The classification result shows *Dusk & Dawn* with a 100% probability, which means that the classifier works correctly. From 17:00 UT, the sky darkened completely, as shown in the all-sky image at $T_1$. The classification result for this period was *No aurora*, which is also correct because this label is a combination of *Clear* and *Cloudy*, as already shown in Fig. 1. The green aurora appeared in the keogram from $\sim$19:30 UT and the classifier recognized it as *Arc* and *Discrete*. At $T_2$, a typical east-west arc was observed in the all-sky image and the classifier detected *Arc* with a probability of 100%. At $T_3$, an auroral breakup occurred, with an active discrete aurora covering most areas of the sky. The classifier detected *Discrete* correctly. After that, the *Diffuse* aurora was detected until 23:00 UT, and a relatively dimmer diffuse aurora was also visible in the keogram and the all-sky image at $T_4$. The classifier was able to detect the aurora almost successfully through the entire episode of the auroral substorm from 19:30 until 23:00 UT. Another substorm after midnight was also detected without any problem.

Interestingly, at 18:30 and 23:30 UT, the classifier detected *Arc* with a probability of $\sim 60\%$, and the faint aurora was visible near the northern limit (upper part) of the all-sky image at these times. These arcs appeared in a much smaller area than the typical aurora seen during substorms; thus, the classifier may not respond with a high probability value. These very faint auroras could be noise in the statistical analysis because sometimes, the classifier responded and sometimes, they did not. Based on this, we defined the auroral appearance as a time when the sum of the probabilities of the *Auroral* classes exceeded 80%. This threshold value was used for the statistical analysis that will be presented in the next section. Although all detections were performed automatically, we eventually created images in the same format as that shown in Fig. 3 for all observation dates over 10 years, and visually confirmed that the classification results were correct to the same level as that demonstrated in Fig. 3.

### Variations of auroral occurrence

**Figure 4:**
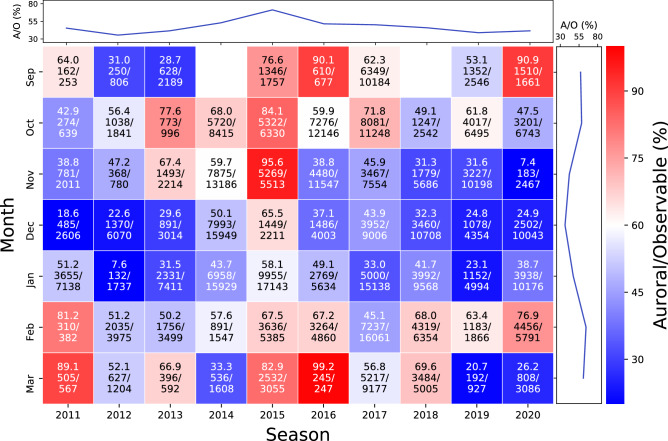
Monthly occurrence rate of aurora for the past 10 years. Each panel shows, from top to bottom, the occurrence rate, number of *Auroral* classes, and number of *Observable* classes. The red panels indicate high occurrence rate, and the blue panels indicate low occurrence rate. The blue line in the upper panel shows annual variation in the occurrence rate obtained by data integration. Similarly, the blue line in the right panel shows monthly variation in the occurrence rate. The occurrence rate was the highest in the 2015 season and tended to be higher in fall and spring than in winter.

Figure 4 shows the distribution of the auroral occurrence rate as a function of month and year over 10 years. The vertical axis is the month and the horizontal axis is the year of optical season, i.e., from September to March in the next year. Again, the auroral occurrence rate is defined as the number of the *Auroral* images divided by that of the *Observable* images. Each square panel filled with colors indicates the occurrence rate, the number of the *Auroral* images, and that of the *Observable* images. While the number of images varies widely from month to month, as it is affected by the length of cloudy periods and the temporal resolution, the occurrence rate is less affected by these factors. In September 2014 and 2018, where the numbers are unavailable, the camera was not operative due to the delay in starting the observation. Throughout the decade, the panel is mostly bluish from November to January, indicating a lower auroral occurrence rate. To make it easier to observe this tendency, we have added the right panel, which shows the monthly variation of the occurrence rate derived by summing the numbers over the decade. The panel clearly demonstrates the decreasing trend of auroral occurrence in the winter solstice. Furthermore, it was found that there was no significant difference between the auroral occurrence rate in the autumn and spring equinoxes and that the graph was nearly lined symmetrical with respect to December. The same method was applied to plot the upper panel, which shows the annual variation of the auroral occurrence rate. It did not peak in the 2013 season, which was when the sunspot number reached its maximum during solar cycle 24, but instead, in the 2015 season, which corresponds to the early declining phase of the solar cycle. On the other hand, the auroral occurrence rate attained its minimum during the 2019 season, which was when the sunspot number reached its minimum.

**Figure 5:**
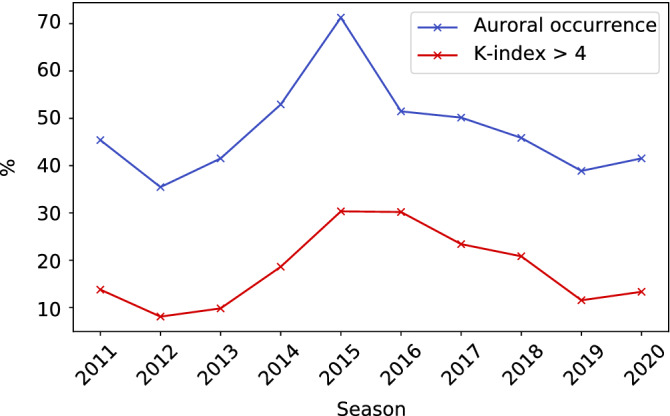
The blue line shows annual variation in the occurrence rate of aurora. The red line shows the percentage of times when the K-index was 4 or higher during clear nights in Tromsø. Years with higher K-index have higher occurrence rates.

Since all the images used in this study were captured in Tromsø, it is likely that local parameters representing the level of geomagnetic disturbances, such as the K-index, rather than macroscopic/global parameters, such as the sunspot number, would correlate well with the auroral occurrence rate. The K-index represents the geomagnetic disturbance in a 10-point scale (0–9) with a three-hour resolution; the higher the K-index, the more likely it is for an aurora to appear. Figure 5 shows the annual variation of the auroral occurrence rate shown in Fig. 4 and the ratio of time when the K-index was above 4 during a local night in Tromsø, i.e., 15–03 UT. Note that only days with more than 100 *Observable* images were included in the calculation. The blue and red lines attained their minimums in the 2012 and 2019 seasons, respectively, and a maximum in the 2015 season, indicating that the level of local geomagnetic disturbance and automatically derived auroral occurrence rate are positively correlated over a long period of 10 years.

**Figure 6:**
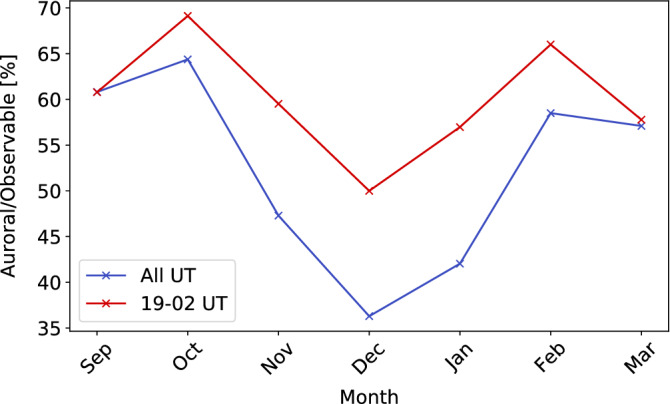
Monthly variation in the occurrence rate of aurora for all UT (blue line) and 19 to 02 UT only (red line). During 19 to 02 UT, the sky is completely dark in all the months from September to March in Tromsø. In both lines, the occurrence rate is the lowest in December, which includes the winter solstice, and it is high near the spring and autumn equinoxes.

Figure 6 shows the monthly variation of the auroral occurrence rate shown in Fig. 4. The blue line covers all UT, while the red line covers only 19–02 UT, i.e., the time when the sky is completely dark from September to March. The occurrence rate of the red line, which is for limited UT periods, is higher than that in the blue line, suggesting that the auroral occurrence rate is lower in the early and later UT. In both the lines, the auroral occurrence rate attains its maximums in October and February and a minimum in December. This means that regardless of the length of the dark hours, the occurrence of auroras is higher in autumn and spring and lower in winter. Especially, the blue line shows a difference of nearly two-fold between the auroral occurrence rates in October and December. It has been pointed out since the beginning of the 20th century that the frequency of magnetic storms is higher near the spring and autumnal equinoxes, and lower around the summer and winter solstices^25^. This effect is often interpreted using the misalignment of the Earth’s rotation and magnetic axes and the 30-day periodicity of the spiral structure of the solar wind, and is well known as the Russell–McPherron (R–M) effect^26^. The monthly variation of the auroral occurrence rate is qualitatively consistent with the characteristics of the R–M effect.

**Figure 7:**
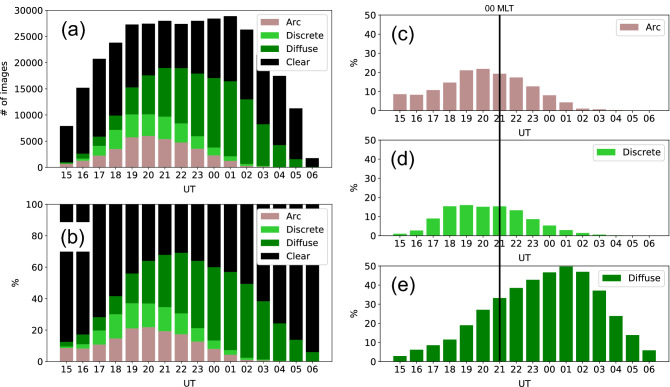
(**a**) Histogram showing the composition of images of *Observable* classes obtained over 10 years. A decrease in the number of images in 15–18 UT and 03–06 UT is due to the difference in the the length of the dark time. (**b**) The composition ratio of the number of images in the *Observable* classes. The occurrence rate of aurora was maximum at 22 UT (01 MLT). (**c**)–(**e**) Composition ratios of panel (**b**) for each *Auroral* class. The occurrence rate of discrete auroras (*Arc* and *Discrete*) peaked before 00 MLT and was almost zero after 01 UT (04 MLT). *Diffuse* was most likely to occur at 01 UT (04 MLT) and the occurrence rate was widely distributed across other UTs.

Figure 7 shows the UT distribution of the *Observable* classes obtained over a period of 10 years. Panel (**a**) shows the number of images, panel (**b**) shows the composition ratio of panel (**a**) for each UT bin, and panels (**c**)–(**e**) show the values of panel (**b**) for each *Auroral* class. In panel (**a**), while more than 25000 images were stably classified as *Observable* in 19–02 UT, the number of the images increased/decreased in 15–18/03–06 UT, respectively. This is due to the monthly change in the length of the dark time. To reduce this effect, the composition ratio of each class in each UT bin is shown in panel (**b**). The lower ratio of the *Auroral* classes to the *Clear* class in the early and late UT was consistent with the suggestion from Fig. 6 that auroras occur less frequently in these UT periods. The ratio of the *Auroral* classes reached its maximum at 22 UT, which roughly corresponds to 01 MLT. This result is consistent with the results of previous studies obtained by visual detection^9–11^. Panel (**b**) shows that discrete auroras are dominant in the evening hours and diffuse auroras are dominant from the magnetic midnight (00 MLT $\sim$ 21 UT) to the morning. The comparison of panels (**c**) and (**d**) shows that the ratio of *Arc* is higher than that of *Discrete* in 15–18 UT, which may be due to the tendency that intense discrete auroras occur after the appearance of an arc-type aurora^23^, as seen in Fig. 3. The *Discrete* auroras had a wide plateau at 18–21 UT ($\sim$21–00 MLT). By contrast, the ratio of *Diffuse* auroras gradually increased from the evening hours, peaked at 01 UT ($\sim$ 04 MLT), and then decreased towards dawn. This trend is consistent with the results of other statistical analyses performed visually and using automated detection techniques^8,14,27^.

### A notification service for auroral activity

**Figure 8:**
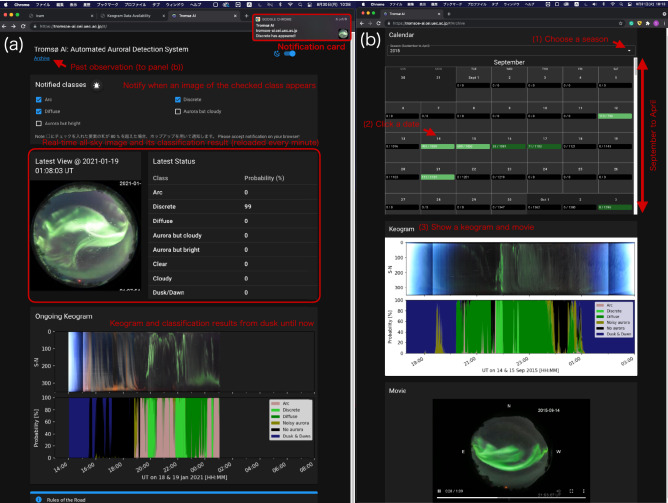
(**a**) Top page of Tromsø AI. In the central part, the all-sky image and the classification result are displayed in real-time. In the lower part, the ongoing keogram from the sunset is displayed in the same format as Fig. 3. A desktop notification appears in the upper right when an image of the classes checked in “Notified Classes” is displayed. Clicking on “Archive” below the page title trans the archive page shown in panel (**b**). (**b**) A user can view a keogram and all-sky movie on any day by selecting a season from the drop-down menu and clicking on a date in the displayed calendar.

The results of the previous section have quantitatively revealed the annual, monthly, and UT variations of the auroral occurrence in Tromsø. However, such overall statistical characteristics cannot be used to understand the auroral appearance at specific moments in the future. To overcome this limitation, we developed “Tromsø AI” (https://tromsoe-ai.cei.uec.ac.jp/), a web application that allows AI to monitor the auroral activity in Tromsø in real-time, instead of the human eye. Panel (a) in Fig. 8 shows the top page at 01:08:03 UT on 19th January 2021. The all-sky image shown in the Latest View shows a dynamic discrete aurora, and its classification result, Latest Status, indicates that the image belongs to the *Discrete* class with 99% probability. This page has a desktop notification feature of the auroral appearance, which sends a notification card shown in the upper right if an image belongs to the checked classes in the box at the top of the page (i.e., if the sum of the probabilities of the checked classes exceeds 80%). Note that a desktop notification must be allowed in both the OS and the browser to use this function. The bottom of the page provides an ongoing keogram in the same format as that in Fig. 3, showing the auroral activity up to that time of the night. In addition, a user can browse all observation results for the last ten years by clicking on the “Archive” link at the bottom of the title. Panel (b) shows the page for the archived data. A user can select a season from the pull-down tab to view the calendar for the corresponding period (from September to April). Clicking on a date in the calendar will display the keogram and movie for that night. Each date in the calendar has a fraction, the numerator of which is the number of images classified as *Auroral* classes and the denominator is the total number of images obtained that night. Days with a high number of *Auroral* images are shown in green color on the calendar.

## Discussion

Figure 5 shows the positive correlation of the auroral occurrence rate with the ratio of the highest K-index time. Both reached their maximum in the 2015 season, which was an early declining phase of the solar activity. The Kp-index, which is a global version of the local K-indices, increases during the phases of declining solar activity^28^, and our results demonstrate that the occurrence of auroras also increases in the early declining phase of the solar cycle. During this early declining phase, the auroral occurrence rate exceeded 70%. In contrast, before the solar maximum and during the solar minimum (the 2019 season), it was $\sim$35%, i.e., half as much as the peak. Figure 6 shows that the auroral occurrence rate tends to be higher in spring and autumn and lower in winter. Compared to the lowest auroral occurrence rate in December, the highest one in October was 65%, showing nearly a two-fold difference. This is most likely due to the R–M effect^26^, which explains why geomagnetic storms are more frequent in the spring and autumn equinoxes and less frequent in the winter and summer solstices. This tendency has already been reported in previous studies^6,29^, but because these previous studies have classified the images by human eye, not all the images could be classified by a stable/unique standard. In this study, since the classification is performed using a computer, the images taken over a 10-year period can be classified by the same standard in a short calculation time.

Panels (c) to (e) in Fig. 7 show the occurrence rate of auroras from each *Auroral* class as a function of UT. For discrete auroras, which consists of *Arc* and *Discrete* auroras, the occurrence rate peaked in the pre-midnight sector and decreased sharply in the morning sector, as shown in previous studies^14,30^. A slightly larger composition ratio of the *Arc* auroras compared with that of the *Discrete* auroras may reflect the fact that arc-type auroras are the main element of auroras that tend to appear under all geomagnetic conditions^31^. The occurrence rate of *Diffuse* peaked in the morning sector, which is also consistent with previous studies^4,14,27,32,33^. Diffuse auroras could originate due to magnetospheric waves, such as chorus^34–37^ and electrostatic electron cyclotron harmonic (ECH) waves^38,39^, the former often being widely distributed from the midnight to noon sector^40^ and the latter from the pre-midnight to morning sector^41^. In panel (e), the occurrence rate of diffuse auroras decreased in the morning sector owing to the effect of sunlight, but their occurrence over a wide range of UT was generally consistent with the distribution of the magnetospheric waves. These results indicate that the large number of the images taken in the last decade were classified successfully. The dependences of the occurrence rate obtained in this study will be useful not only for data analysis purposes, but also for a wide range of applications, from planning of campaign observations to personal travel.

The website shown in Fig. 8 was created to promote citizen science, and to provide easy access to past observation data. This website provides instant updates on the appearance of auroras in Tromsø. Furthermore, if this system is installed at multiple observatories, the broader distribution of auroras can be obtained in real-time. Moreover, unlike professional optical instruments, digital cameras can obtain color information. It has recently been suggested that the energy of precipitating electrons can be estimated qualitatively based on the color of a digital image^5^, which can help enhance the contribution of citizen scientists to professional space science. The current auroral notification system can help the citizen scientist know the appearance of aurora and start their observation promptly. If they are able to capture more optical data with less efforts by using the system, the threshold for participating the auroral science would become lower. Therefore, the system introduced here is a promising approach to reveal new features of aurorae and improve our understanding of the processes underlying these phenomena.

## Methods

### Optical observation in Tromsø, Norway

Since 2011, Nikon digital cameras (D5000, D5100 and D7200) have been capturing all-sky images almost every night from September to March with a temporal resolution of less than one minute at Ramfjordmoen Research Station in Tromsø, Norway ($69.6^\circ \hbox {N}$, $19.2^\circ \hbox {E}$); the station is operated by the UiT - the Arctic University of Norway. These images were not originally planned to be used for a statistical analysis of the auroras, as they were obtained to evaluate weather conditions during the acquisition of atmospheric temperature/wind observations using a sodium LIDAR^42–45^ and multi-wavelength observations of auroras using a photometer^46–48^. The digital cameras used were D5000 during 2010–2014, D5100 during 2015, and D7200 during 2016–2021. A Sigma 4.5 mm f/2.8 EX DC HSM Circular Fisheye lens was used. The temporal resolution, exposure duration, and ISO sensitivity are 1 min, 15 s, and 2500 during 2011–2013, and 30 s, 8 s, and 3200 during 2014–2021, respectively. Although the shooting settings varied from year to year, the colors and shapes of the stars, clouds, and auroras in the JPEG images did not change significantly. The images taken by the digital camera are uploaded to the following URL: https://www.isee.nagoya-u.ac.jp/~eiscat/obs/d5000/html/sky_image.html. The original image size is $4496 \times 3000$ pixels, but the uploaded image has been downsampled to $722 \times 480$ pixels. In addition, the uploaded images are labeled with texts giving the time and directions of the image. By classifying the uploaded images, we conducted the statistical analysis and developed the real-time notification system.

### Setting of training and definition of image classes

The classification algorithm is based on the previous studies^20,22^, with minor modifications to apply it for noisy (i.e., contaminated auroral) images. One of our objectives is to detect an aurora in color all-sky images, including cloudy ones. The previous study^22^ showed that the ResNet-50 model classifies auroras into seven classes with high accuracy, but an image without auroras was not considered. Here, the ResNet-50 means a residual network having 50 layers. The ResNets have skip connections between hidden layers, which makes it difficult to decrease accuracy even when the number of layers increases. The other study^20^ proposed a design of six classes that can classify actual auroral data properly, although grayscale images were the target of the classification. Based on the results of these previous studies, we reconstructed the eight classes by renovationg their definitions based on the latter study^20^ and classified images using the same model as the former study^22^ (ResNet-50). To evaluate the auroral occurrence more accurately, we defined new classes of auroras with cloudy and moonlit conditions, which were not considered in the latter study^20^. The details of the definition of each class are described below. The percentage of the field of view occupied by clouds was estimated based on visual inspection. The threshold value for bright and dark was not determined using the solar elevation angle, but by visually checking whether the darkness level was the same as that at midnight on a new moon day. This brightness standard was applied not only to the effects of sunlight, but also to those of moonlight. All images were downsampled to a resolution of 128 $\times$ 128 pixels as in the former study^22^, as indicated by the white dashed square in the *Arc* panel in Fig. 1, before being used for training. Other pre-processing was not performed.

After the pre-processing, we fine-tuned the pre-trained weights on the ImageNet dataset^49^ using the training data. The batch size and epoch were 128 and 4, respectively. The stochastic gradient descent (SGD) was used as the optimizer, and the learning rate and momentum were given arguments of 0.001 and 0.9, respectively. The program of the training was written using Keras^50^.

#### Arc

Images with one or two auroral arcs extending in an east-west direction, a dark background, and less than 20% of the field of view occupied by clouds. These auroras often appear the prior to the onset of substorms.

#### Discrete

Images with non-*Arc* discrete auroras, a dark background, and less than 20% of the field of view occupied by clouds. These are intense auroras that often appear with the auroral breakup.

#### Diffuse

Images with diffuse auroras (including pulsating auroras), a dark background, and less than 20% of the field of view occupied by clouds.

#### Aurora but cloudy (AC)

Images with auroras, a dark background, and more than 20% of the field of view occupied by clouds.

#### Aurora but bright (AB)

Images with auroras and a bright background, regardless of the cloud cover.

#### Clear

Images with no aurora, a dark background, and less than 20% of the field of view occupied by clouds.

#### Cloudy

Images with no aurora, a dark background, and more than 20% of the field of view occupied by clouds.

#### Dusk and Dawn (DD)

Images with no aurora and a bright background, regardless of the cloud cover. This class includes not only images brightened by sunlight, but also those illuminated by moonlight.

The training and validation datasets manually labeled from the above definitions can be downloaded from the following URL: https://tromsoe-ai.cei.uec.ac.jp/~nanjo/public/dataset/, which can be used by the readers to evaluate the performance of the classifier with auroral images taken at different places by using different cameras.

Despite the detailed classification, some images do not belong to any of the above classes. We did not use such images in the training because they do not contain the aurora and therefore would not affect the task of extracting aurora-containing images (*Auroral* classes + *Noisy aurora*) from a large number of the regularly captured images.

## Data Availability

The color all-sky images used in this study are available at https://www.isee.nagoya-u.ac.jp/~eiscat/obs/d5000/html/sky_image.html. Images for a past specific date in the past can be obtained by giving an 8-digit number in the format of YYYYMMDD. For example, images on 26 January 2022 are available at https://www.isee.nagoya-u.ac.jp/~eiscat/obs/d5000/html/pre20220126.html. The training and validation datasets used in this study are available at https://tromsoe-ai.cei.uec.ac.jp/~nanjo/public/dataset/. The K-index at Tromsø is available at https://flux.phys.uit.no/Kindice/Listindex.html.

## References

[1] MacDonald EA (2018). New science in plain sight: Citizen scientists lead to the discovery of optical structure in the upper atmosphere. Sci. Adv..

[2] Gallardo-Lacourt B (2018). A statistical analysis of steve. J. Geophys. Res. Space Phys..

[3] Palmroth M (2020). Citizen scientists discover a new auroral form: Dunes provide insight into the upper atmosphere. AGU Adv..

[4] Nishimura Y (2020). Diffuse and pulsating aurora. Space Sci. Rev..

[5] Nanjo S (2021). Periodicities and colors of pulsating auroras: Dslr camera observations from the international space station. J. Geophys. Res. Space Phys..

[6] Nevanlinna H (1995). Auroral observations in finland-visual sightings during the 18th and 19th centuries. J. Geomagn. Geoelectr..

[7] Eddy JA (1976). The maunder minimum. Science.

[8] Nevanlinna H, Pulkkinen TI (2001). Auroral observations in finland: Results from all-sky cameras, 1973–1997. J. Geophys. Res. Space Phys..

[9] Sheret, M. *Analysis of Auroral Observations, Halley Bay, 1959, British Antarctic Survey Scientific Reports* Vol. 37 (British Antarctic Survey, London, 1963).

[10] Blackie, J. *Analysis of Auroral Observations, Halley Bay, 1960, British Antarctic Survey Scientific Reports* Vol. 40 (British Antarctic Survey, London, 1964).

[11] Blundell, G. *Analysis of Auroral Observations, Halley Bay, 1961 and 1962, British Antarctic Survey Scientific Reports* Vol. 48 (British Antarctic Survey, London, 1967).

[12] Yang Q, Liang J, Hu Z, Zhao H (2012). Auroral sequence representation and classification using hidden Markov models. IEEE Trans. Geosci. Remote Sens..

[13] Syrjäsuo M, Donovan E (2002). Analysis of auroral images: Detection and tracking. Geophysica.

[14] Syrjäsuo MT, Donovan EF (2004). Diurnal auroral occurrence statistics obtained via machine vision. Ann. Geophys..

[15] Rao J, Partamies N, Amariutei O, Syrjäsuo M, van de Sande KEA (2014). Automatic auroral detection in color all-sky camera images. IEEE J. Sel. Top. Appl. Earth Observ. Remote Sens..

[16] Wang Q (2010). Spatial texture based automatic classification of dayside aurora in all-sky images. J. Atmos. Solar Terr. Phys..

[17] Fu, R., Li, J., Gao, X. & Jian, Y. Automatic aurora images classification algorithm based on separated texture. In *2009 IEEE International Conference on Robotics and Biomimetics (ROBIO)*, 1331–1335. 10.1109/ROBIO.2009.5420722 (2009).

[18] Zhong Y, Huang R, Zhao J, Zhao B, Liu T (2018). Aurora image classification based on multi-feature latent dirichlet allocation. Remote Sens..

[19] Jia Z, Han B, Gao X, Zha H, Chen X, Wang L, Miao Q (2015). 2dpcanet: Dayside aurora classification based on deep learning. Computer Vision.

[20] Clausen LBN, Nickisch H (2018). Automatic classification of auroral images from the Oslo auroral Themis (oath) data set using machine learning. J. Geophys. Res. Space Phys..

[21] He, K., Zhang, X., Ren, S. & Sun, J. Deep residual learning for image recognition. In *2016 IEEE Conference on Computer Vision and Pattern Recognition (CVPR)*, 770–778. 10.1109/CVPR.2016.90 (2016).

[22] Kvammen A, Wickstrøm K, McKay D, Partamies N (2020). Auroral image classification with deep neural networks. J. Geophys. Res. Space Phys..

[23] Karlsson T (2020). Quiet, discrete auroral arcs–observations. Space Sci. Rev..

[24] Lessard, M. R. *A Review of Pulsating Aurora* 55–68 (American Geophysical Union (AGU), 2012).

[25] Cortie, S. A. L. Sun-spots and terrestrial magnetic phenomena, 1898–1911: The cause of the annual variation in magnetic disturbances. *Mon. Notices R. Astron. Soc.* **73**, 52–60. 10.1093/mnras/73.1.52 (1912).

[26] Russell CT, McPherron RL (1973). Semiannual variation of geomagnetic activity. J. Geophys. Res..

[27] Partamies N (2017). Occurrence and average behavior of pulsating aurora. J. Geophys. Res. Space Phys..

[28] Rangarajan GK, Iyemori T (1997). Time variations of geomagnetic activity indices kp and ap: An update. Ann. Geophys..

[29] Silverman SM (1992). Secular variation of the aurora for the past 500 years. Rev. Geophys..

[30] Gillies DM (2014). A survey of quiet auroral arc orientation and the effects of the interplanetary magnetic field. J. Geophys. Res. Space Phys..

[31] Partamies N, Juusola L, Whiter D, Kauristie K (2015). Substorm evolution of auroral structures. J. Geophys. Res. Space Phys..

[32] Jones SL, Lessard MR, Rychert K, Spanswick E, Donovan E (2011). Large-scale aspects and temporal evolution of pulsating aurora. J. Geophys. Res. Space Phys..

[33] Bland EC, Partamies N, Heino E, Yukimatu AS, Miyaoka H (2019). Energetic electron precipitation occurrence rates determined using the syowa east superdarn radar. J. Geophys. Res. Space Phys..

[34] Nishimura Y (2010). Identifying the driver of pulsating aurora. Science.

[35] Jaynes AN (2013). Pulsating auroral electron flux modulations in the equatorial magnetosphere. J. Geophys. Res. Space Phys..

[36] Kasahara S (2018). Pulsating aurora from electron scattering by chorus waves. Nature.

[37] Hosokawa K (2020). Multiple time-scale beats in aurora: Precise orchestration via magnetospheric chorus waves. Sci. Rep..

[38] Fukizawa M (2018). Electrostatic electron cyclotron harmonic waves as a candidate to cause pulsating auroras. Geophys. Res. Lett..

[39] Fukizawa M (2020). Pitch-angle scattering of inner magnetospheric electrons caused by ech waves obtained with the arase satellite. Geophys. Res. Lett..

[40] Li W (2009). Global distribution of whistler-mode chorus waves observed on the themis spacecraft. Geophys. Res. Lett..

[41] Ni B (2011). Global distribution of electrostatic electron cyclotron harmonic waves observed on themis. Geophys. Res. Lett..

[42] Tsuda TT (2011). Fine structure of sporadic sodium layer observed with a sodium lidar at Tromsø Norway. Geophys. Res. Lett..

[43] Tsuda TT (2013). Decrease in sodium density observed during auroral particle precipitation over Tromsø Norway. Geophys. Res. Lett..

[44] Nozawa S (2014). Variations of the neutral temperature and sodium density between 80 and 107 km above Tromsø during the winter of 2010–2011 by a new solid-state sodium lidar. J. Geophys. Res. Space Phys..

[45] Tsuda TT (2015). A sporadic sodium layer event detected with five-directional lidar and simultaneous wind, electron density, and electric field observation at Tromsø Norway. Geophys. Res. Lett..

[46] Adachi K (2017). Evaluation of a method to derive ionospheric conductivities using two auroral emissions (428 and 630 nm) measured with a photometer at Tromsø (69.6$$^{\circ }$$N). Earth Planets Space.

[47] Nozawa S (2018). A new five-wavelength photometer operated in Tromsø (69.6$$^\circ$$N, 19.2$$^\circ$$E). Earth Planets Space.

[48] Kawamura Y (2020). Estimation of the emission altitude of pulsating aurora using the five-wavelength photometer. Earth Planets Space.

[49] Deng, J. *et al.* Imagenet: A large-scale hierarchical image database. In *2009 IEEE Conference on Computer Vision and Pattern Recognition*, 248–255. 10.1109/CVPR.2009.5206848 (2009).

[50] Chollet, F. *et al.* Keras. https://github.com/fchollet/keras (2015).

